# Tannic Acid as a Versatile Template for Silica Monoliths Engineering with Catalytic Gold and Silver Nanoparticles

**DOI:** 10.3390/nano12234320

**Published:** 2022-12-05

**Authors:** Irina Postnova, Yury Shchipunov

**Affiliations:** 1 Institute of Chemistry, Far-East Department, Russian Academy of Sciences Vladivostok, 690022 Vladivostok, Russia; 2 Institute of High Technologies and Advanced Materials, Far-Eastern Federal University, 690922 Vladivostok, Russia

**Keywords:** tannic acid, template, sol-gel, monolithic silica, silver nanoparticles, gold nanoparticles, bionanocomposite, surface plasmon resonance, catalyst

## Abstract

Tannic acid in alkaline solutions in which sol-gel synthesis is usually performed with tetraethoxysilane is susceptible to various modifications, including formation of reactive radicals, oxidation under the action of atmospheric oxygen, self-association, and self-polymerization. Here, a precursor with ethylene glycol residues instead of ethanol was used, which made it possible to synthesize bionanocomposites of tannic acid and silica in one stage in neutral media under normal conditions without the addition of acid/alkali and organic solvents. Silica was fabricated in the form of optically transparent monoliths of various shapes with 2–4 nm pores, the radius of which well correlated with the size of a tannic acid macromolecule in a non-aggregated state. Polyphenol, which was remained in pores of silica matrix, served then as reducing agent to synthesize in situ gold and silver nanoparticles. As shown, these Au@SiO_2_ and Ag@SiO_2_ nanocomposites possessed localized surface plasmon resonance and high catalytic activity.

## 1. Introduction

**Figure 1:**
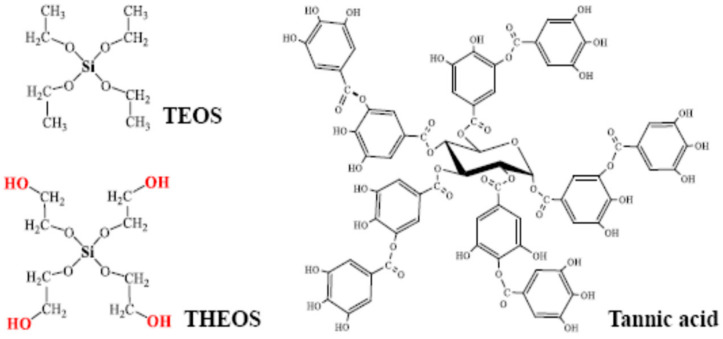
Structural formulas of tetraethoxysilane (TEOS), tetrakis(2-hydroxyethyl)orthosilicate (THEOS) and tannic acid (TA).

Tannins, which belong to polyphenols, are ubiquitous terrestrial substances of the Earth, after cellulose, lignin and hemicellulose. Their functional role in plants consists of defense against micro-organisms, fungi and herbivores. This is explained by the broad spectrum of their biological activities: antibacterial, antiviral, anticancer, antimutagenic, antioxidant and UV-protective. The beneficial properties of polyphenols attract much attention [1,2,3,4]. Furthermore, tannins possess some practically important physico-chemical properties. They are due to numerous phenolic OH-groups. Hydroxyl groups are carried by gallic acid residues, as can be seen from the structural formula of tannic acid (TA) shown in Figure 1. They tend to form hydrogen bonds, covalent bonds via Michael addition/Schiff base reactions and chelate complexes with metal cations. These properties of tannins are used to bind proteins, polysaccharides and metal salts [5,6,7,8,9,10,11]. 

Non-toxic biodegradable natural biopolymers containing numerous hydroxyl groups can be an alternative to templates based on traditionally used non-green surfactants and block copolymers for regulating sol-gel processes and the mesoporous structure of composite materials [12,13,14,15,16,17,18,19]. This was shown first with the example of polysaccharides, which accelerated reactions and manipulated the silica structure [20,21,22]. The approach was then extended to regulate the poorly controlled sol-gel synthesis of titanium dioxide [23,24]. As shown recently, polyphenols containing numerous hydroxyl groups are also suitable for the fabrication of silica bionanocomposites. This was demonstrated for the first time by Gao and Zharov who could synthesize monodisperse spherical particles of about 200 nm in diameter in an alcoholic solution of TA using a modified Stöber method [25]. The polyphenol served as a non-surfactant pore-regulating template. The pore size, depending on the concentration of TA, varied in the range of 6–13 nm. In [26,27], silica microparticles with similar sizes and porosity were similarly synthesized. Boesel and Sadeghpour et al., who studied five tannins, pointed out their great morphogenetic potential. It consists, in their opinion, in the formation of a supramolecular skeleton, which governs the mesoporosity of silica [28]. 

Tannins are important not only as a template determining the structure of the synthesized bionanocomposites, but also as their functional component [4,6,9,29,30]. It was shown in [31] that the polyphenol left in the pores of the silica conferred antimicrobial activity. It also imparted the sorption of copper (II) ions [30] and proteins, including enzymes [25,32]. TA remaining after synthesis in the pores of the mesoporous SiO_2_ matrix made it possible to reduce and prepare catalytically active silver nanoparticles [33]. Silica was also functionalized by attaching tannins to amino groups on their surface through the Michael addition/Schiff base reaction. The attached polyphenols then served to prepare highly active catalysts containing noble metal nanoparticles [34,35] adsorbents for wastewater treatment from dyes [36] and protein sorption [37]. 

Sol-gel synthesis is frequently carried out with tetraethoxysilane (TEOS) (Figure 1) [38,39,40,41]. Its disadvantage is the low rate of condensation reaction in the neutral region. To accelerate it, acid or alkali is added as a catalyst for the sol-gel processes. The acidic and especially alkaline regions are not optimal for TA. The alkali leads to the dissociation of hydroxyl groups (Figure 1), charging of molecules, formation of radicals and oxidation under the action of atmospheric oxygen, self-association into aggregates, and self-polymerization [28]. Carrying out sol-gel processes with TEOS in the neutral region is practically impossible and restricts the possibilities of the common approach [13,14,18]. 

Hoffmann et al. suggested replacing the alcohol residues in TEOS with ethylene glycol, thus preparing a new tetrakis(2-hydroxyethyl)orthosilicate (THEOS) precursor (Figure 1) [42,43]. They demonstrated that the THEOS hydrolysis along with the condensation reactions proceeded rapidly in the neutral pH region without slowing down the sol-gel process [42,43]. Owing to the precursor with ethylene glycol residues, bionanocomposites with proteins in the entire pH range, including acidic, neutral, and alkaline regions, were fabricated [14,44]. THEOS also made it possible to immobilize enzymes at their optimal pH values, which are usually in the vicinity of the neutral region [45,46]. It was recently used for the first time by our group for the synthesis of a monolithic silica on a tannin template without the addition of alkali or acid [47]. 

Herein, the synthesis of silica in the neutral region on a TA template was performed for the first time. It is shown that in this case a monolithic optically transparent bionanocomposite is formed without changing the color of the polyphenol. When the synthesis was accomplished in an alkaline medium in [28], the color changed to dark brown. Unchanged color means that, if TA is oxidized by atmospheric oxygen, it is to a minimal extent. Retention of the reducing properties of TA made it possible to synthesize silver and gold nanoparticles in mesopores, which had a high catalytic activity.

## 2. Materials and Methods

### 2.1. Materials 

Tannic acid (TA), silver nitrate (99.9%) and sodium borohydride were purchased from Sigma-Aldrich. Hydrogen tetrachloroaurate and 4-nitrophenol were “chemically pure”. Tetraethoxysilane (TEOS) was obtained from ABCR company. It was used together with ethylene glycol (99%, Acros Organics) to synthesize tetrakis(2-hydroxyethyl)orthosilicate (THEOS) as previously described in [20,48]. Structural formulas of silica precursors and TA are shown in Figure 1. Aqueous solutions were prepared with deionized water obtained from a Rios-Di Clinical water purification system (Merck, Darmstadt, Germany). 

### 2.2. Methods 

Bionanocomposites TA@SiO_2_ were synthesized by admixing 5–50 wt.% THEOS into an aqueous solution with 1–5 wt.% TA. The mixture was thoroughly stirred for 1–2 min and left at the room temperature for a few days. TA@SiO_2_ obtained as a monolith was transferred for 30 min into a freshly prepared solution of silver nitrate or hydrogen tetrachloroaurate; the concentrations of which varied from 0.01 to 1 mM. The optimal concentration range, determined after testing catalytic activity, was 0.1–0.3 mM. To synthesize Ag and Au nanoparticles, samples were immersed for 30 min in 8 mL AgNO_3_ or HAuCl_4_ solution protected against light sources. The samples, after removal of excess solutions from their surface, were transferred to a thermostated cabinet for 30 min, where they were kept at 80 °C. They were then transferred to water to wash out from TA that was removed almost completely after 5–6 water changes. To carry out the catalytic reaction, washed Au@SiO_2_ and Ag@SiO_2_ were immersed in a 10 mL stirred solution containing 0.5 mM 4-nitrophenol and 10 mM NaBH_4_. The course of the catalytic process was monitored by spectrophotometrically. 

The absorption spectra were recorded on a UV-2250 spectrophotometer (Shimadzu, Kyoto, Japan) in the wavelength range of 300–700 nm. The silica was placed in a holder for solid samples, and the solutions, in a glass cuvette 1 cm thick. Morphology was studied using a scanning electron microscope (SEM) Hitachi S-5500 (Tokyo, Japan). A Hitachi HT7700 (Tokyo, Japan) was used as a transmission electron microscope (TEM). The examination was carried out in the bright field mode at an accelerating voltage of 100 kV. Porosity characteristics were evaluated from nitrogen sorption measurements by means of an ASAP 2010 apparatus (Micromeritics) in the relative pressure range of *P/P*_0_ = 0.05–0.99 at 77 K after outgassing the powdered sample at 323 K under vacuum. The pore size distribution was calculated from the desorption branch of an isotherm by using the Barrett–Joyner–Halenda (BJH) model. Surface area and volume of micro-mesopores were calculated by the BET method. 

## 3. Results and Discussion 

### 3.1. Sol-Gel Synthesis 

The formation of the silica begins with the hydrolysis reaction of the precursor added to the TA solution, according to the nucleophilic substitution of an ethylene glycol by the hydroxyl group according to the following reaction [14,38,39]:

Si(-OR)_4_ + nH_2_O → (HO-)_n_Si(-OR)_4-n_ + nHO-R (1)

where R is the residue of ethylene glycol OH-CH_2_-CH_2_-OH, and *n* ≤ 4. With the appearance of a reactive silanol group, the precursor molecule is drawn into condensation reactions. There are two opportunities [14,38,39]. In one case, the reaction proceeds with the participation of two silanol groups:

(RO-)_4−n_Si(-OH)_n_ + (HO-)_n_Si(-OR)_4−n_ → (OH)_n−1_ (RO-)_4−n_Si-O-Si(-OR)_4−n_ OH)_n-1_ + H_2_O, (2)

which leads to the formation of a dimer in which two silicon atoms are connected by a covalent bond through the Si-O-Si oxygen bridge. The second reaction proceeds between the hydrolyzed and initial precursor molecules according to the equation:

Si(-OR)_4_ + (HO-)_n_Si(-OR)_4−n_ → (RO-)_3_Si-0-Si(-OR)_4−n_ OH)_n−1_ + HO-R, (3)

As a result of this reaction, a dimer is also formed, in which the silicon atoms are linked in the same manner through an oxygen bridge. If there are other hydroxyl groups in the molecule, then the dimer is involved into further condensation reactions. This results in the formation of a polymer chain, which may be linear, branched or cyclic. 

Reactions (1)–(3) proceeded at room temperature and at a pH value of solutions in the range of 5–6. Addition of acid, alkali and organic solvent was not needed. The rate of sol–gel processes and the formation of TA@SiO_2_ bionanocomposite depended significantly on the concentrations of both the precursor and TA. A monolithic silica begun to form at a THEOS concentration of 10 wt.%. An increase in the precursor content from 10 to 50 wt.% sharply accelerated the gelation of solutions. A sol to gel transition was caused the formation of a three-dimensional network from polysilicic acids and TA. Time varied from 1–2 days to several minutes. In the latter case, a slight heating of the solutions was observed. The polyphenol also influenced the transition to the gel state, but to a lesser extent, slowing down the processes. 

**Figure 2:**
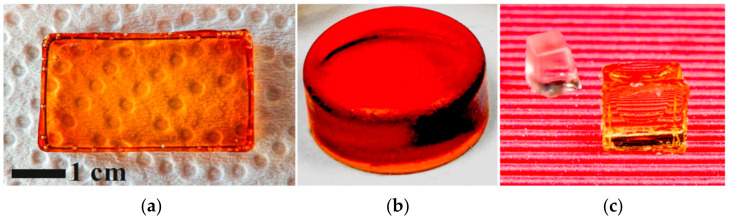
Pictures of samples of the TA@SiO_2_ bionanocomposite synthesized in solutions with TA, in the form of a plate 8 mm thick (**a**), a disk (**b**), and cubes (**c**). Picture (**c**) on the left shows a SiO_2_ sample after washing off the TA.

The entrapment of TA into the silica matrix was evidenced by the characteristic color. It varied from golden (light yellow-brown) to intense reddish-brown, which depended on the polyphenol concentration and sample thickness. The color of the initial solutions practically was not changed after gelation. It also remained almost unchanged after drying TA@SiO_2_, which indicated the absence of changes (oxidation) of the polyphenol. The advantage of this version of sol-gel method is that it can be used to synthesize bionanocomposites with TA of various shapes: in the form of plates, disks, rods, cubes, etc. They can be seen in Figure 2. 

The optical transparency of prepared TA@SiO_2_ samples should be noted (Figure 2). There were no cracks or significant curl in them. This should be attributed to the advantages of sol-gel synthesis on a TA template. When using polysaccharides and proteins, optically transparent bionanocomposites can only be synthesized in exceptional cases [14,20,21,22,44]. The crucial point in their case is the sol-gel transition. In its course, optically transparent initial solutions become opalescent and then opaque. Differences in optical properties are evidence for discrepancy in the porous structure discussed below. 

It should be noted that TA was quite easily extracted with water up to complete removal from the synthesized TA@SiO_2_. This was obvious from the disappearance of the color. As an example, Figure 2c shows a picture of such a sample. Washout provided evidence on the formation of open, interconnected pores in a silica matrix. 

In the vast majority of studies in which TA was used as a template, the syntheses were carried out according to the modified Stöber method [26,27,28,32,33,36]. In this case, sufficiently concentrated ammonia solutions of alcohol are taken, in which the hydrophobic precursor of TEOS (Figure 1) is soluble, and the process itself is conducted with vigorous stirring. Under such conditions, only microparticles are obtained [49,50]. The method allowing for the formation of monolithic silica was used only in the work [31], but the bionanocomposites were synthesized in it in the form of a powder. The authors, in contrast to our approach, traditionally took TEOS (Figure 1) by adding acid or alkali to the reaction mixtures and performing the processes under heating. They did not perform synthesis in neutral media. The studies showed that the use of TEOS does not make it possible to obtain silica monoliths with biopolymers [13,14,20,21,22,51]. This, as demonstrated here in the case of TA, can only be done with the THEOS precursor (Figure 1 and Figure 2). 

### 3.2. Bionanocomposite Structure

**Figure 3:**
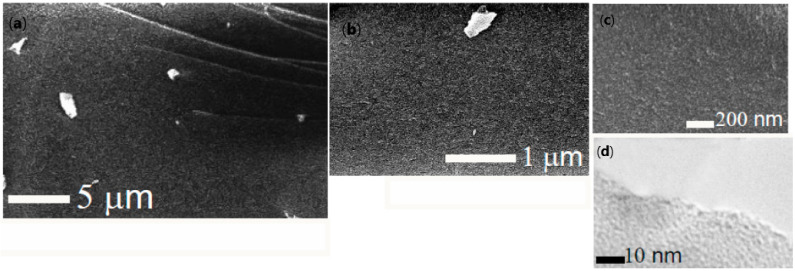
SEM (**a**–**c**) and TEM (**d**) images of the same sample of SiO_2_. They were taken at different magnifications. TA was preliminarily removed by washing in water. The sol-gel synthesis was performed by admixing 20 wt% THEOS to a 2 wt% TA solution.

A series of images taken at different magnifications using a SEM is shown in Figure 3a–c. The surface looks smooth and homogeneous. Any pores larger than tens of nanometers are not seen. This differs from that usually observed in the case of mesoporous silica fabricated by the common sol-gel method. Some particles seen in the images were obtained when the sample was cut in the course of preparation for the examination.

The absence of large pores is also confirmed by the observation under TEM (Figure 3d). In this case, pores with a size of few nanometers are only distinguishable. Individual isolated pores can be seen, but their number is limited. Most of them are connected to each other, which confirms the conclusion made above from the results of washing TA@SiO_2_ from polyphenol. 

**Figure 4:**
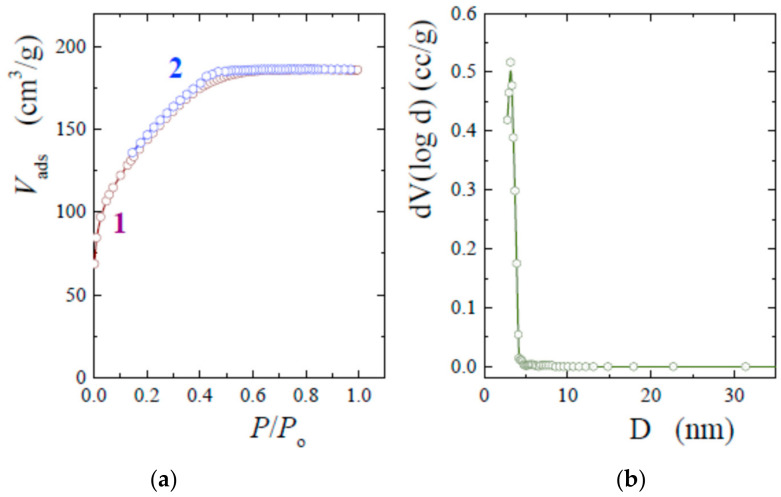
(**a**) Adsorption–desorption isotherms from nitrogen sorption for a sample synthesized by adding 20 wt% THEOS into a solution with 1 wt% TA at ambient conditions. (1) Adsorption and (2) desorption branches. (**b**) BJH pore size distribution calculated from the desorption branch.

To better characterize the silica porosity, nitrogen sorption measurements were applied. The samples were preliminarily washed out from TA in deionized water. Nitrogen adsorption–desorption isotherms are shown in Figure 4a. The concavity of the curves with respect to the abscissa axis and reaching a plateau at *P*/*P_o_* → 1 makes it possible to refer them to the Type I isotherm [52]. This type occurs in the case of microporous materials. A small hysteresis loop provides a means for the calculation of the size distribution of pores. The graph is shown in Figure 4b. The maximum, as can be seen from the plot, is at 3.1 nm. The peak, well-defined and highly narrow, indicates an extremely narrow size distribution. An essential feature is that it correlates well with the size of the TA molecule, which is 2.2 × 1.8 × 1.2 nm [25]. The correlation points to the important role of TA in the formation of TA@SiO_2_: polyphenol serves as a template that regulates the sol–gel process and determines the structure of the bionanocomposite in full measure. 

The conclusion is in line with that in [25,26,32]. The authors also pointed out the important role of TA in the sol-gel synthesis. However, the porosity of the obtained bionanocomposites differs markedly. According to the data presented in the articles [25,26,27,32,33], the pore diameter varies from 4.2 to 13 nm. At the same time, 4.2 nm was obtained only in [33], while the authors in [27,32] gave 8.4 and 9.4 nm. It was assumed that such large sizes are explained by the aggregation of TA macromolecules. The pore diameter in our case is equal to 3.1 nm (Figure 4b). A 3-fold increase in the content of TA in the reaction mixture did not lead to a notable change in their size. It somewhat increased, but did not exceed 3.3 nm. In the work [26], in which the effect of polyphenol concentration on silica porosity was considered in detail, a significant change in the pore diameter from 4.3 to 9.9 nm was noted. 

Rough agreement in the sizes of the pores (3.1 nm) and the TA macromolecule (2.2 × 1.8 × 1.2 nm) indicates the absence of polyphenol aggregation in our experiments. Much the same situation was previously noted in the case of cyclodextrins embedded in the silica matrix in a similar way [51]. In the case when the surface of TA macromolecules was covered with a silica molecular shell, which prevented polyphenol association, the pores after the sol–gel synthesis in [30] were equal to 3.8 nm. 

Discrepancies in the results and conclusions regarding TA aggregation are explained by differences in the conditions of the sol-gel synthesis. In [25,26,27,32,33] it followed the method developed by Stöber [49], according to which the TEOS (Figure 1) and polyphenol were brought into an alcohol solution of ammonia of a sufficiently high concentration. In alkaline media, TA transfers into a charged state due to the dissociation of hydroxyl groups in the gallic acid residues (Figure 1). Furthermore, polyphenol can be oxidized, self-assemble and self-polymerize [2,7,9,29,53]. All of these factors together could provide the association of TA macromolecules with each other, which is a valid explanation for large pores in the silica matrix. Syntheses with THEOS were conducted in a neutral solution. TA in it is rather stable and does not undergo notable changes that take place in alkaline media. Apparently, syntheses at different pH of aqueous solutions were the reason for the discrepancy from the literature data in the porosity of synthesized TA@SiO_2_. 

### 3.3. Synthesis of Gold and Silver Nanoparticles Embedded into Silica 

The ability of TA to reduce noble metals has been known for a long time [54], and has recently been used for the synthesis of nanoparticles [9,10,29]. The property was retained when TA was included in the silica matrix, making it possible to synthesize nanosized particles of silver, gold, and palladium in pores in situ [33,34,35]. This was used by us to functionalize the synthesized TA@SiO_2_. In this case, the fabrication of nanoparticles could be started immediately after the formation of the silica, since the polyphenol was already included in the pores. 

**Figure 5:**
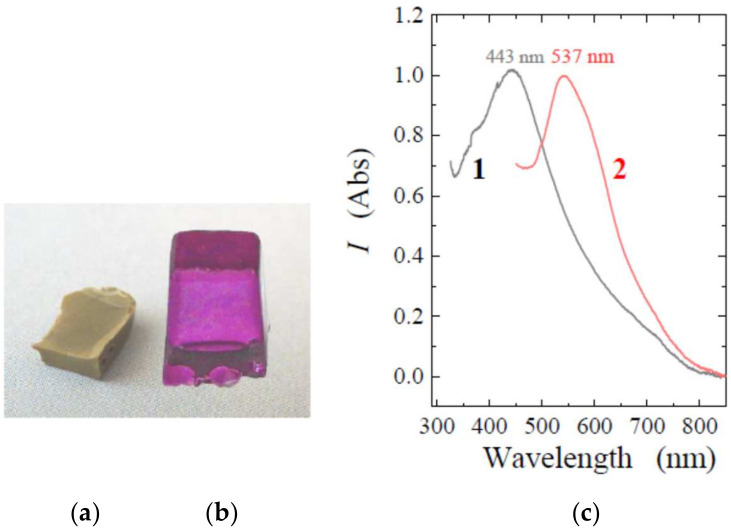
(**a**) Silica coated with excess silver. The sample was obtained on TA@SiO_2_ without washing from polyphenol after the sol-gel synthesis. (**b**) Silica containing gold nanoparticles in the internal volume. The sample was synthesized after preliminary washing in a solution containing 0.1 wt% TA. (**c**) UV-vis spectra characterizing the presence of Ag (1) and Au (2) nanoparticles in silica matrix. The polyphenol was preliminarily removed from the samples by washing before examination.

However, early experiments showed poor control of the noble metal reduction process. Silver and gold concentrated mainly on the surface of the silica monolith, forming a rather dense coating (Figure 5a). The limitation was the high concentration of TA used as the template in the sol-gel synthesis. The outflow of polyphenol, which appeared immediately after the contact of a sample with a silver or gold salt solution, restricted the counter diffusion into the pores being in the interior of silica matrix, involving them into the complex and then reducing them. Over time, the access began to be hindered by the filled pores, and coating formed on the surface.

To obtain a more uniform distribution of nanoparticles in the bulk of the silica matrix and improve control over the process, excess amounts of TA were washed out of synthesized TA@SiO_2_ bionanocomposites with water. To create a certain concentration of TA in TA@SiO_2_, improve control over the process and obtain reproducible results, the washed samples were placed in a dilute TA solution in which they were kept for 1–3 days. For synthesis of nanoparticles, they were then transferred into a solution of a silver or gold salt. 

The nanoparticle formation could become available from a gradual change in color to purple, as seen in the case of Au (Figure 5b). In addition, it was confirmed spectrophotometrically. The spectra are shown in Figure 5c. Characteristic UV-Vis absorption is due to the phenomenon known as localized surface plasmon resonance [55,56]. As obvious from absorbance spectra presented in Figure 5c, bandwidths are observed at around 443 and 537 nm for Ag and Au nanoparticles, respectively. This is correlated with the literature data [56,57,58]. The absorption peaks at 443 and 537 nm are typical of spherical nanoparticles. Their aggregation or/and change of shape results in red-shifting and broadening in the bandwidth [56,57,59]. It could occur partially in the case of gold nanoparticles because of shoulder seen in spectrum 2. 

**Figure 6:**
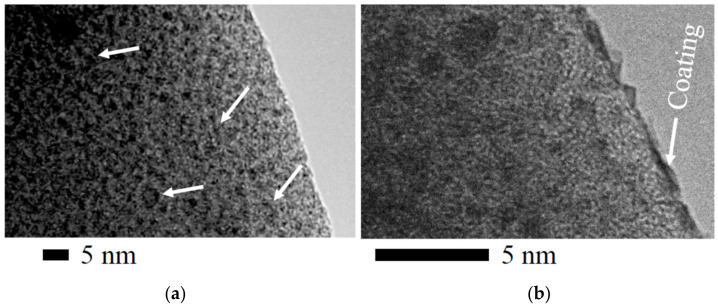
TEM images of an Au@SiO_2_ bionanocomposite sample taken at two different points. (**a**) Arrows show contacting or merged nanoparticles located in adjacent pores inside the silica matrix. (**b**) The arrow shows the coating formed on the surface of the silica. The bionanocomposite was prepared by dipping TA@SiO_2_ containing 0.1 wt.% TA into a solution with 0.2 mM HAuCl_4_. Polyphenol was washed out after the synthesis.

The formation of nanosized particles inside the silica matrix was also confirmed by means of TEM. Some images are presented in Figure 6. The size of most of the nanoparticles, as can be seen, is in the range of 2–3 nm, i.e., it does not exceed the pore diameter determined using nitrogen sorption measurements (Figure 4b). On this basis, it can be concluded that the nanoparticles were actually formed and located in the pores of the silica matrix. 

In some cases, one can see structures apparently formed from contacted or merged particles. They are shown by arrows in Figure 6a. Their cross-sectional diameter also does not exceed 3 nm. The arrow in Figure 6b points in elongated nanoparticles that were formed predominantly on the surface of the silica. Such nanoparticles are few in number. Therefore, the whole blocking of the internal volume from the entry of gold and silver ions did not occur. The process of reduction and formation of nanoparticles could proceed both on the surface and inside the bionanocomposite. 

An undoubted advantage of nanosized particles fabrication in the pores of a silica matrix is the limitation of their size by the volume in which they are formed. They cannot exceed it. When the pore is filled, continuation is possible only through one-dimensional growth, i.e., propagation into neighboring pores. The presence of contacting or merged particles (Figure 6a) indicates that in some cases this happened, but not often. The limitation could be due to the low concentration of TA and salts optimized in such a way as to exclude the formation of a solid coating on the surface. 

The formation of nanoparticles in pores hinders their coalescence, which is usually observed during the synthesis in solutions. To prevent it, along with the reducing agent, the introduction of stabilizing additives is needed [60,61]. The restriction for the movement of nanoparticles localized in the pores eliminates coalescence, which ensures their stabilization without the addition of stabilizers. 

After the synthesis and the fabrication of a silica matrix doped with nanosized particles of a certain size, a functional bionanocomposite was obtained. Its application is possible in various fields. In particular, the manifestation of localized surface plasmon resonance (Figure 5c) is of interest for the development of photonic materials and sensors [61,62,63,64]. Another possibility exists of using these bionanocomposites in catalysis, since the catalytic activity of nanosized gold and silver is well known [61,62,63,64]. It has been verified in the present work. 

### 3.4. Catalytic Properties 

**Figure 7:**
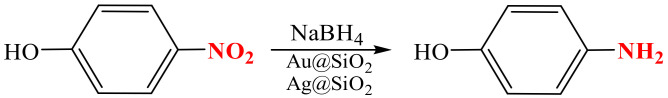
Catalytic reduction of 4-nitrophenol with the formation of 4-aminophenol.

**Figure 8:**
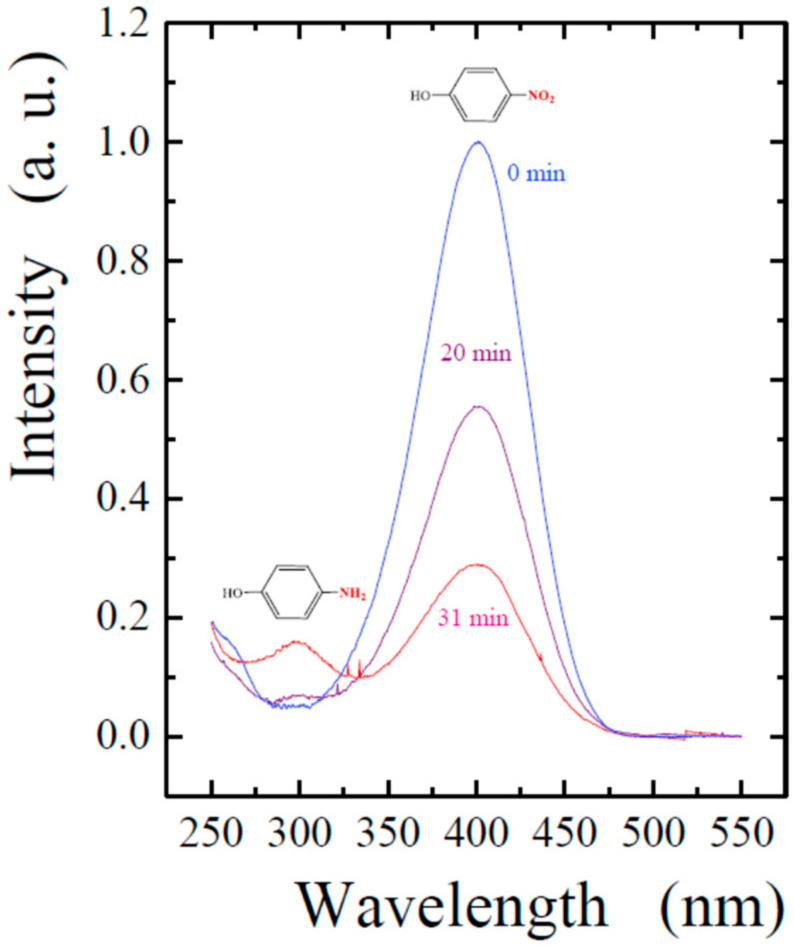
UV-vis spectra of the reaction mixture before the start of the process (0 min, spectrum of 4-nitrophenol) and after a number of time intervals (shown next to the curves) after the start of the catalytic reduction reaction. The initial concentration of 4-nitrophenol was 0.5 mM, NaBH_4_, 10 mM. The bionanocomposite was prepared by dipping TA@SiO_2_ containing 0.1 wt.% TA into a solution with 0.2 mM AgNO_3_.

The well-known reduction of 4-nitrophenol was chosen as a model reaction. Nitroaromatic compounds are stable chemicals. Even strong reducing agents do not have a noticeable effect on them under normal conditions. The situation is changed in the presence of nanosized particles of noble metals [65,66]. They catalyze the reduction of the nitro group to the amino group at room temperature according to the reaction (Figure 7):

Sodium borohydride is usually taken as the reducing agent. The progress of the reaction is monitored spectrophotometrically. 4-Nitrophenol is determined by the characteristic peak at 400 nm (Figure 8). Reduction with borohydride leads to the formation of 4-aminophenol, which is revealed by a new peak at 295 nm. Its intensity is almost an order of magnitude less. The difference can be seen visually by the color of the solutions. Before the start of the reaction, they are colored yellow, and as 4-nitrophenol is reduced and 4-aminophenol is formed discoloration occurs. 

**Figure 9:**
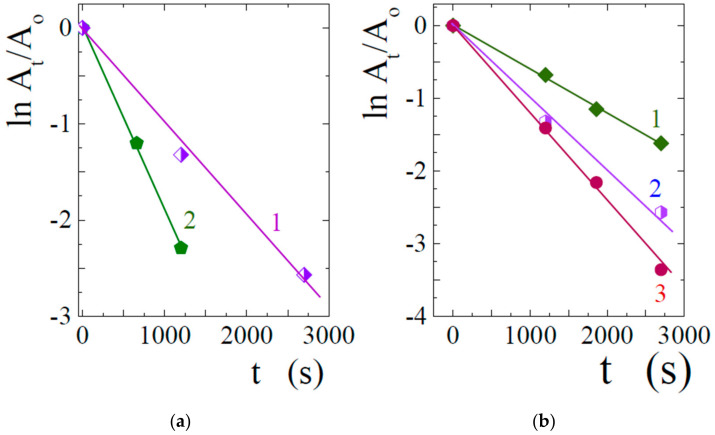
Time-dependent changes of the normalized optical absorbances at 400 nm (Figure 7). The stock solutions contained 0.5 mM 4-nitrophenol and 10 mM NaBH_4_. Bionanocomposites were prepared by dipping TA@SiO_2_ containing 0.1 wt.% TA into a 10 мл solution with (**a**) 0.2 mM AgNO_3_ (line 1) and 0.2 mM HAuCl_4_ (line 2); (**b**) 0.4 (line 1), 0.2 (line 2) and 0.1 (line 3) mM AgNO_3_.

The course of the reaction is usually monitored by the gradual decrease in the intensity of the peak at 400 nm. As an illustration, the spectra of the studied solutions are shown in Figure 8, measured at different time intervals after the start of the catalytic process. Since it was carried out with a significant excess of the reducing agent, it can be assumed that the reaction rate did not depend on the concentration of 4-nitrophenol. Therefore, the kinetics of its reduction can be considered as pseudo first-order in accordance with the equation:

ln (A_t_/A_0_) = −k_ap_ t, (4)

where A_0_ and A_t_ are, respectively, the initial absorbance at 400 nm and the absorbance measured at time t after the start of the process, k_ap_ is the apparent rate constant. Graphs in coordinates ln (A_t_/A_0_) − t for a number of studied samples are shown in Figure 9. All the experimental points would fall on a straight line, from the tangent of the slope angle of which the numerical value -k_ap_ can be found. 

Figure 9a shows the results of testing photocatalytic activity. Samples containing gold and silver nanoparticles were synthesized under similar conditions. In addition, TA@SiO_2_ silicas were of the same size and shape, which had the form of a slightly truncated cone. The bottom surface had a size of 5 × 5 mm, and the edge of the cone was 7 mm. Such silica was synthesized in silicone molds with identical cells to obtain ice cubes in refrigerator freezers. It was possible to simultaneously synthesize up to 40 identical samples from the same initial solution prepared for the sol-gel synthesis. They can be seen in Figure 2c and Figure 5b. Catalytic activity was tested under strictly identical conditions. One of the Au@SiO_2_ or Ag@SiO_2_ samples was placed in 10 mL of a solution containing 0.5 mM 4-nitrophenol and 10 mM NaBH_4_. The process time was varied to study the kinetics of the reduction.

The apparent rate constants of the 4-nitrophenol reduction reaction determined from the slope of straight lines in Figure 9a in the case of Au@SiO_2_ and Ag@SiO_2_ turned out to be 0.0019 and 0.00094, respectively. Gold nanoparticles can be seen to have a higher catalytic activity than silver. 

**Figure 10:**
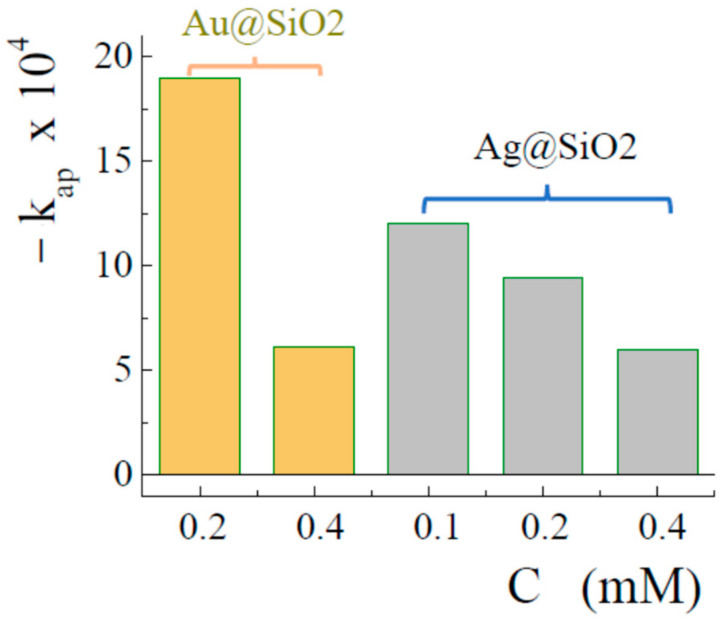
Apparent reduction rate constants of 4-nitrophenol for samples prepared in solutions with different gold and silver salt contents.

The catalytic activity depended highly on the conditions of sample preparation. In particular, noticeable differences were found in the properties of bionanocomposites prepared in solutions with different concentrations of gold and silver salts. This can be seen from Figure 9b. Figure 10 summarizes the data on the apparent rate constants of the 4-nitrophenol reduction reaction for Au@SiO_2_ and Ag@SiO_2_ synthesized in solutions with different salt contents. As can be seen, in both cases there is the same trend, which consists in a decrease in the catalytic activity for the samples synthesized in more concentrated solutions. A possible explanation may lie in the formation of larger nanoparticles on the surface of the silica matrix. They are less catalytically active and, in addition, can block the pores (Figure 6b), affecting the accessibility for substances. 

In summary, we have successfully synthesized bionanocomposites of silica with TA using the green sol-gel method. It is based on the use of a precursor, which, unlike existing approaches, does not need the acidification/alkalization of solutions and the addition of organic solvents. The fast process can be conducted in neutral aqueous solutions, which are optimal for TA. The suggested method made it possible to synthesize new silica bionanocomposites with TA, which cannot be fabricated using the approaches suggested in the literature. They are fabricated in the form of optically transparent monoliths with a extremely narrow pore size distribution, which are comparable in size to the TA macromolecule. It has been shown that one of the promising applications of novel bionanocomposites can be the fabrication of catalysts. Using TA as a reducing agent, the reduction in situ of noble metals in the pores of the silica matrix was carried out. As shown, they possessed high catalytic activity. 

## Data Availability

Not applicable.
